# Machine Learning and Its Applications for Protozoal Pathogens and Protozoal Infectious Diseases

**DOI:** 10.3389/fcimb.2022.882995

**Published:** 2022-04-28

**Authors:** Rui-Si Hu, Abd El-Latif Hesham, Quan Zou

**Affiliations:** ^1^ Institute of Fundamental and Frontier Sciences, University of Electronic Science and Technology of China, Chengdu, China; ^2^ Yangtze Delta Region Institute (Quzhou), University of Electronic Science and Technology of China, Quzhou, China; ^3^ Genetics Department, Faculty of Agriculture, Beni-Suef University, Beni-Suef, Egypt

**Keywords:** artificial intelligence, machine learning, protozoal parasite, image detection, public health, host-parasite interaction, drug and vaccine discovery

## Abstract

In recent years, massive attention has been attracted to the development and application of machine learning (ML) in the field of infectious diseases, not only serving as a catalyst for academic studies but also as a key means of detecting pathogenic microorganisms, implementing public health surveillance, exploring host-pathogen interactions, discovering drug and vaccine candidates, and so forth. These applications also include the management of infectious diseases caused by protozoal pathogens, such as *Plasmodium*, *Trypanosoma*, *Toxoplasma*, *Cryptosporidium*, and *Giardia*, a class of fatal or life-threatening causative agents capable of infecting humans and a wide range of animals. With the reduction of computational cost, availability of effective ML algorithms, popularization of ML tools, and accumulation of high-throughput data, it is possible to implement the integration of ML applications into increasing scientific research related to protozoal infection. Here, we will present a brief overview of important concepts in ML serving as background knowledge, with a focus on basic workflows, popular algorithms (e.g., support vector machine, random forest, and neural networks), feature extraction and selection, and model evaluation metrics. We will then review current ML applications and major advances concerning protozoal pathogens and protozoal infectious diseases through combination with correlative biology expertise and provide forward-looking insights for perspectives and opportunities in future advances in ML techniques in this field.

## Introduction

Machine learning (ML), a core field under AI, is an important technology in the domain of bioinformatics (Larranaga et al., 2006). When facing various large and complex data requiring processing, ML can leverage sophisticated algorithms and establish effective models to find meaningful information from massive complex datasets (Xu and Jackson, 2019). As a step forward in science technology, the marriage between mathematics and computer science in ML has shown substantial promise and has been applied to many scientific fields, such as biomedicine (Goecks et al., 2020), phytology (Sperschneider, 2020; Sun et al., 2020; Wang et al., 2021), and microbiology (Qu et al., 2019; Peiffer-Smadja et al., 2020a; Goodswen et al., 2021a). Previously, most of these studies demonstrated the advent of high-throughput technologies that led to increased interest in the use of ML approaches and the combination of a plethora of omics data to conduct in-depth data mining. However, ML has also created new inroads, moving from more considerable theoretical research to practical applications, such as biological-image analysis (Litjens et al., 2017; Moen et al., 2019), disease prediction (Zou et al., 2018; Lee et al., 2021), and diagnostic microbiology (Sinha et al., 2018; Peiffer-Smadja et al., 2020b). Particularly, with the worldwide COVID-19 pandemic in recent years, relevant studies have advanced the development of AI-driven health technologies to solve relevant biological problems of microbial infections (Schwalbe and Wahl, 2020). The causative agents causing infectious diseases include various types of microorganisms, such as bacteria, viruses, fungi, and protozoans. More recently, Goodswen et al. (2021a) reviewed ML application in microbiology, with a significant focus on pathogenic microorganisms, such as predicting drug and vaccine candidates, tracking disease outbreaks, exploring microbial interactions, and detecting pathogens. To date, ML applications have shown a broad spectrum of prospects in every microbiology discipline, including bacteriology, mycology, virology, and parasitology.

The protozoan parasites, belonging to the research category of parasitology, represent an important class of single-celled eukaryotes within the kingdom of microorganisms. A list of the most common and important protozoan parasites and their relative data information is summarized in **Table 1**. These protozoan parasites are infamous due to their ability to infect humans and animals and lead to corresponding diseases. Here, three representative protozoal diseases are exemplified owing to high mortality and morbidity risk in cases of infection. The first example is malaria caused by *Plasmodium* parasites, leading to an estimated 229 million people infected worldwide in 2019, with the latest WHO report indicating an estimated number of deaths of up to 409,000 (WHO, 2019); the second example is Chagas disease caused by *Trypanosoma cruzi*, capable of affecting 6-7 million people worldwide in 2017 and causing an estimated 7,900 deaths (Collaborators, 2018); the third example is toxoplasmosis caused by *Toxoplasma gondii*, resulting in infection of one-third of the world’s population (most are asymptomatic): this disease is known to cause life-threatening human encephalitis (Elsheikha et al., 2021). Other protozoan parasites are also important in the context of public health risks, which have been documented in detail in the WHO program (WHO, 2019). For of all these protozoal diseases, medicinal treatment is the only solution to alleviate the symptoms of infections; however, the emergence of drug resistance is rapidly spreading and persisting, and there are no commercial vaccines for protozoans yet except that RTS,S/AS01 (RTS,S) has recently been approved by WHO for the prevention of *P. falciparum* malaria in children (Laurens, 2020)

**Table 1:** An overview of main human protozoan parasites and the available genome links.

Causative agent	Taxonomic group	Caused disease	Genome link^†^
*Acanthamoeba*	Amoebozoa	Acanthamoeba keratitis	https://amoebadb.org/amoeba/app/downloads/
*Entamoeba histolytica*	Amoebozoa	Amoebiasis	https://amoebadb.org/amoeba/app/downloads/
*Babesia* spp.	Apicomplexa	Babesiosis	https://piroplasmadb.org/piro/app/downloads/
*Cyclospora cayetanensis*	Apicomplexa	Cyclosporiasis	https://toxodb.org/toxo/app/downloads/
*Cryptosporidium* spp.	Apicomplexa	Cryptosporidiosis	https://cryptodb.org/cryptodb/app/downloads/
*Plasmodium* spp.	Apicomplexa	Malaria	https://plasmodb.org/plasmo/app/downloads/
*Toxoplasma gondii*	Apicomplexa	Toxoplasmosis	https://toxodb.org/toxo/app/downloads/
*Leishmania* spp.	Kinetoplastida	Leishmaniasis	https://tritrypdb.org/tritrypdb/app/downloads/
*Trypanosoma brucei*	Kinetoplastida	African sleeping sickness	https://tritrypdb.org/tritrypdb/app/downloads/
*Trypanosoma cruzi*	Kinetoplastida	Chagas disease	https://tritrypdb.org/tritrypdb/app/downloads/
*Giardia lamblia*	Metamonada	Giardiasis	https://giardiadb.org/giardiadb/app/downloads/
*Trichomonas vaginalis*	Metamonada	Trichomoniasis	https://trichdb.org/trichdb/app/downloads/

**^†^** Refer to the links provided by Aurrecoechea et al. (2017). The download link can access to the corresponding species in database.

Examples of ML’s usefulness, such as image recognition-based pathogen detection, protozoal disease prediction, and the ability to solve various complex or nonlinear disease problems, could aid scientists in building effective diagnostic methods and developing new intervention measures. Given the advancement of ML, including evolutionary DL algorithms (Lecun et al., 2015), protozoal infectious diseases caused by protozoal pathogens, causing great global concern regarding public health issues, are part of a growing number of objects that use ML as an analytical tool to address relative biological problems. This review will present a brief description of ML, including deep neural networks, serving as background knowledge, and providing a survey and overview of current ML applications and advances in protozoal pathogens and protozoal diseases.

## Machine Learning

### Example Basic Workflow

**Figure 1:**
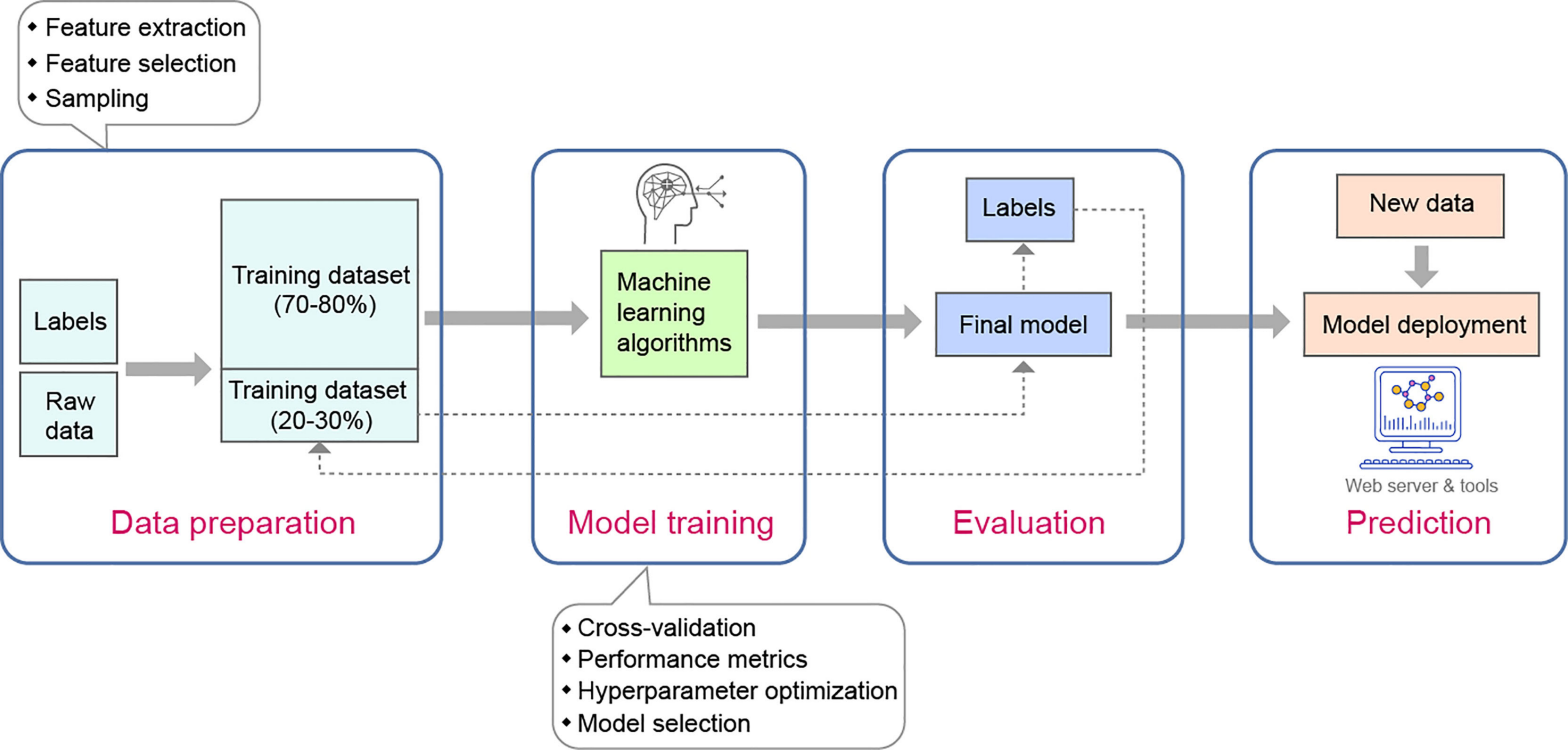
Example schematic workflow of constructing a machine learning predictor (supervised learning approach). The overall flue contains four steps, namely, data preparation, model training, evaluation, and prediction. Step1: data are preprocessed to ensure suitability for machine learning and are split into training and test cohorts. The preprocessed data are characterized (i.e., numerical vector with labels) by feature extraction method and the optimal features are charactered by feature selection methods (such as by MRMD software). Step2: A type of machine learning algorithm (e.g., SVM, RF, or neural network) is chosen based on the data to be used and the desired task, and the models are trained on training dataset. Step3: model performance is evaluated using the test dataset through cross-validation and by means of metrics such as ROC or accuracy. During this step, hyperparameter optimization (or hyperparameter tuning) is usually performed to determine the right combination of hyperparameters that maximizes the model performance: for example, the maximum depth allowed for a decision tree algorithm and the number of trees contained in a random forest algorithm. Step4: an optimal model is chosen as the final model and packaged appropriately for users (e.g., online webserver for prediction or scripts for local use).

In the ML pipeline, a variety of data types can be used as input materials, such as numerical data, categorical data, time-series data, and textual information. These data types can be interconverted according to the actual need. Prior to starting with model entailing and data training, data preprocessing, such as data normalization and discarding missing and duplicate values, should be performed to ensure the reliability of the results in the subsequent analysis. The degree to which datasets must be preprocessed for ML varies, depending on the choice of model and the nature of the research problem of interest. Raw input data, such as biological sequences (DNA, RNA, and protein/peptide sequences), are often multi-dimensional and may contain irrelevant or redundant data; thus, feature selection and feature extraction are typically needed so that the learning accuracy and the result comprehensibility will be improved. After preparation, datasets can typically be split into training and testing cohorts (often with 70-80% of the data for training and 20-30% for testing) (Gholamy et al., 2018). Data for training cohort are used to build a prediction model and data for test cohort are used to evaluate the performance of a model. In addition, several other signs of progress also need to be considered to determine the most suitable model and make the model practically useful: for example, cross-validating the performance of a model, accessing the uncertainty regarding a given prediction, and alternatively performing hyperparameter optimization (or hyperparameter tuning) in order to determine the appropriate combination of hyperparameters that maximizes the model performance. A representative example of a basic ML workflow is shown in **Figure 1**.

### Feature Extraction and Feature Selection

In the complete ML workflow, feature extraction (FE) and feature selection (FS) represent two commonly used dimensionality reduction approaches and play pivotal roles in determining the final prediction results (Zebari et al., 2020). FE needs to transfer the existing features into a lower-dimensional space with more robust pattern recognition capability (Cai et al., 2018). Taking protein classification as an example, sequences must be characterized, that is, the sequence information can be converted into a numerical vector following the program’s strategies. The main method of feature representation of amino acid sequences is mainly based on the principles of amino acid composition (single peptide, dipeptide, tripeptide), physicochemical properties, position specificity, conservation, amino acid substitution, secondary structure, and so forth. The commonly used tools include iFeature (Chen et al., 2018) and iLearn (Chen et al., 2021). Unlike FE, FS needs to select a subset of the existing features without a transformation for an original feature. According to the relationship with learning methods, FS algorithms are typically grouped into three types: filter, wrapper, and embedded methods (Chandrashekar and Sahin, 2014). Filter methods (e.g., Pearson’s correlation and Chi-square) are generally used as a preprocessing step, which uses criteria not involving any ML algorithm and does not consider the impact of a selected feature subset on the performance of a given algorithm (Liu and Motoda, 1998; Guyon and Elisseeff, 2006). Regarding performance advantages, filter methods are fast and highly effective, especially for selecting subsets with a large number of features (Talavera, 2005; Sánchez-Maroño et al., 2007). In comparison, wrapper methods leverage the intended predictive model algorithms to select the optimal feature subset, which enables better performance than filter methods (Talavera, 2005). For example, MRMD 2.0 (He et al., 2020) developed previously by our group is a typical wrapped FS tool, which gathers different feature sorting methods and uses PageRank algorithm, as well as performing five-fold-cross-validation through the incremental FS strategy and random forest classifier in order to obtain the optimal feature combination. In the comparison test, the performance of MRMD 2.0 is better than that of filter methods. Moreover, compared to filter and wrapper methods, embedded methods such as using L1 (LASSO) regularization and decision tree perform FS through the training of an algorithm in parallel and combine the respective advantages of filter and wrapper methods (Lal et al., 2006; Bolón-Canedo et al., 2013; Chandrashekar and Sahin, 2014).

### Learning Tasks

ML tasks can be organized into three types: supervised, unsupervised, and semisupervised learning. In a supervised learning task, training data have both features and labels, which are assigned to a prespecified algorithm (e.g., classification or regression) for training, and then to predict an output or target for unlabeled datasets, such as evaluating disease risk based on the known clinical information. In contrast, an unsupervised learning task such as k-means cluster analysis (Ghahramani, 2004) is an exploratory process in nature without the correct label, defined target, and output, but it allows the ML model to discover the similarities and differences in unlabeled datasets. Semisupervised learning is a learning paradigm that simultaneously involves labeled and unlabeled examples to perform certain learning tasks and is a type of ML method that sits between supervised and unsupervised learning (Zhu and Goldberg, 2009). The primary goal of semisupervised learning is to construct a better learning procedure by harnessing unlabeled data when compared to only labeled data. For example, a semisupervised ML approach developed by Ashdown et al. (2020) can establish a cell-based screen model based on labeled and unlabeled parasite images, and the hidden labels are predicted on all unlabeled data using trained models. This method contributes to discriminating diverse parasite morphologies and detecting morphological outliers at different lifecycle stages of the malaria parasite.

### Commonly Used Algorithms

A variety of ML algorithms in supervised and unsupervised learning tasks exist. In microbiological studies (Qu et al., 2019), SVM, NB, RF, and k-NNC are extensively used algorithms. When facing intractable classification problems, SVM can find the most effective means of separating multidimensional space data into two categories (Gonzalez et al., 2005); NB classifies data on the basis of Bayes’ theorem and the independence assumptions between the features (Rish, 2001). RF consists of multiple randomized decision trees and predicts by aggregating the average of the output from diverse trees (Biau and Scornet, 2016). k-NNC implements data classification based on the sample’s similarity (sometimes called distance or closeness) to nearby data points (Peterson, 2009). In practical applications, the type of ML algorithm used typically depends on the type of actual problem being solved, the type of variable number, and the type of trained model that best suits the application, which may decide the predictive results.

Apart from the conventional algorithms mentioned above, neural networks have been considered among the most prolifically and fruitfully used ML algorithms in recent years. Neural networks, also known as artificial neural networks, are a subset of ML and the heart of DL. A neural network is a mathematical model that uses a structure similar to the synaptic connections of brain neurons to process various information and contains multiple processing layers, i.e., an input layer, one or more hidden layers, and an output layer, which consist of interconnected nodes (so-called artificial neurons) (Lecun et al., 2015). A layer of the input node is capable of taking advantage of various source materials (e.g., text, image, or numerical data) and sending them into hidden layers of the network. The hidden layers abstract the representations of the input data to another dimensional space to show more abstract and nonlinear representations. Eventually, data from the output layer result in the desired outcomes.

The main difference between artificial neural networks and DL lies in the scale and complexity of the network layer used. A neural network composed of more than three layers (including input and output layers) can be regarded as a DL algorithm. A previous review in Min et al. (2017) detailed various DL architectures and the research advances in bioinformatics, including deep neural networks, convolutional neural networks, recurrent neural networks, and other emergent architectures. These architectures have been widely applied in many research fields, including omics, biomedical imaging, and biomedical signal processing.

In general, compared to conventional ML algorithms, DL has both advantages and disadvantages. The advantages are that DL bears strong learning ability, wide-coverage, and good portability; the disadvantages are enormous computing power, high hardware cost, and complex model design. In the practical application, which algorithm to choose mainly depends on the size of the data and the intended purpose to achieve.

### Model Evaluation Methods and Metrics

Different ML algorithms bear their own merits, but a suitable algorithm can be obtained by evaluating and comparing different models and by calculating the performance indicators. In this regard, the confusion matrix is a very popular approach when evaluating metrics in binary and multiclass classifications (Kulkarni et al., 2020). It is assumed that the number of occurrences in terms of both positive (P) and negative (N) samples exist in two states, actual and predicted classes. The output “TP” is true positive, which indicates that the number of positive examples is classified correctly. Similarly, the output “TN” is true negative and represents the number of negative samples classified correctly. The term “FP” represents false positive, that is, actual negative samples that are incorrectly classified as positive; the term “FN” indicates false negative samples, namely, actual positive samples are incorrectly classified as negative. Regardless of the type of ML models used, it is crucial to estimate the performance of models by metrics. There are six frequently used metrics, i.e., accuracy, precision, sensitivity (recall), specificity, F-score, and MCC. On the basis of the TP, TN, FP, and FN counts in the model evaluation, their equations are defined by the acquired counts, and the commonly used evaluation metrics were formulated as follows:


(1) $$Accuracy=\frac{TP+TN}{TP+TN+FN+FP}$$

(2) $$Precision=\frac{TP}{TP+FP}$$

(3) $$Sensitivity\left(recall\right)=\frac{TP}{TP+FN}$$

(4) $$Specificity=\frac{TN}{TN+FP}$$

(5) $$F-score=\left(1+\beta^{2}\right)\times \frac{\mathrm{Precision}\times Recall}{\beta^{2}\times \left(Precision+Recall\right)}$$

(6) $$MCC=\frac{TP\times TN-FP\times FN}{\sqrt{\left(TP+FN\right)\times \left(TP+FP\right)\times \left(TN+FP\right)\times \left(TN+FN\right)}}$$

Accuracy [equation (1)] is the number of correct predictions divided by the total number of input samples. It is worth noting, however, predictive accuracy may generate misleading results if used with unbalanced datasets (i.e., datasets in which one class significantly outweighs another class). As an example, a highly unbalanced dataset possibly exists in the classification of infected patients and normal patients based on clinical X-ray images (Bridge et al., 2020). A typical dataset may contain 99% normal pixels (uninfected) and 1% abnormal pixels (infected). The test for infection prediction might give an accuracy of 99%, which is an overly optimistic inflated and unreliable result, suggesting certain limitations of accuracy as a performance indicator. Instead, MCC [equation (6)] considers positive and negative elements ratio in some classification tasks, which confers tremendous advantage in evaluating unbalanced datasets than accuracy (Chicco and Jurman, 2020).

Precision [equation (2)] is calculated as the ratio between true positives and all positives. Recall [equation (3)], also called sensitivity, refers to the ratio between true positive samples correctly classified in the data to all real positive samples. In classification tasks, precision and recall are crucial yet misunderstood as two performance metrics. The choice of whether to use precision or recall relies on the class of questions being asked. For example, the determination of whether an imaged cell is infected with a parasite is sensitive to incorrect classification of a parasite-infected cell image (positive sample) as a parasite-uninfected cell image (negative sample). In terms of this, a number of parasite-infected cells must be sure to be classified as positive samples during the experiment; hence, precision is the preferred metric in evaluation. If only positive samples are detected in tests, the recall rate should be calculated with regards to parasite-uninfected cell images that are classified as positive samples. Indeed, precision and recall accommodate each other. When a model shows better precision but a lower recall rate, which indicates that the model is accurate in classifying samples as positive samples, it can only classify a small number of positive samples. When a model displays higher recall but a lower precision rate, the model classifies the majority of samples as positives, but many false positives can exist in the test. To comprehensively weight precision and recall, the F-score metric [equation (5)] is introduced to evaluate many kinds of ML models. In the formula of F-score, the value of *β* > 1 means that recall is more important than precision, and vice versa. When *β* = 1, i.e., the standard F1-score, which is the harmonic mean of the precision and recall, both performance metrics are considered equally crucial during evaluation.

Moreover, another extensively used evaluation metric in classification processes is the ROC curve, which is plotted with TPR versus FPR, or sensitivity (recall) versus 1-specificity, where TPR or sensitivity is on the Y-axis and FPR or 1-specificity is on the X-axis (Mandrekar, 2010). On the ROC curve, the point near the upper left of the plot represents the critical value with higher sensitivity and specificity. The AUC value acts as a summary of ROC and represents the measure or degree of separating different classes (Fawcett, 2006). Only AUC value > 0.5 or even near 1 indicate that the model or classifier achieves good performance in the evaluations and vice versa.

## Machine Learning Applications for Protozoal Pathogens and Protozoal Infectious Diseases

### Literature Search Strategy

To illustrate the broad utility of ML techniques, we searched PubMed, IEEE Xplore, and Google Scholar databases using the search terms “genus or disease name + machine learning or deep learning or neural networks” (e.g., *Plasmodium* or malaria + machine learning or deep learning or artificial intelligence) for published studies up to August 25, 2021. Because many studies may exist in some applications, we searched representative references from selected articles to enumerate more relevant applications. In total, more than 500 articles were obtained from our search results, and the majority of them were related to malaria parasite (63%), followed by two *Trypanosoma* (13%, and then *Toxoplasma* (8%). However, only 6% of published studies reported ML applications for other protozoal pathogens. In the following sections, we focus on reviewing and discussing ML’s applications in pathogen detection, public health surveillance, host-parasite interaction, drug discovery, omics, and vaccine discovery.

### Pathogen Detection

Establishing time-saving and accurate diagnostic method is crucial in the surveillance, prevention, and control of parasitic diseases. Traditionally, wet-laboratory experiments for molecular diagnoses, such as PCR and real-time PCR, which can directly detect the parasite’s nucleic acid molecule in samples, are highly sensitive for molecular identification. Although these technologies outperform pathogen detection, microscopy methods for diagnostic parasitology offer time savings, low cost, and simplicity advantages. Additionally, microscopy methods are appropriate for point-of-care detection of parasites using blood smears and environmental samples without an available diagnostic laboratory. During the process of microscopy detection, a large number of images often need to be analyzed by health workers; therefore, ML would act as a powerful tool for parasite detection based on image classification (parasite-infected and uninfected cell images). Although all protozoan parasites transition through complicated life cycle stages, each stage differs greatly in morphology and size. Thus, image-based morphology analysis can not only detect the presence of pathogens in microscopic images but also differentiate parasites from diverse lifecycle stages. According to the literature investigated here, the prospects of automating parasite detection using ML methods have aroused broad interest from many researchers owing to their significant advantages. Currently, ML methods are increasingly applied to the detection of various protozoal pathogens, including *Plasmodium* in particular, along with other parasitic protozoans, such as *Toxoplasma*, *Babesia*, *Trypanosoma, Cryptosporidium*, and *Giardia*. Representative papers detailing ML-related methods for parasite image recognition and publicly available diagnostic tools are summarized in **Tables 2**, **3**, respectively.

**Table 2:** Representative artificial intelligence applications for protozoal pathogen detection in publications.

Author	Image acquisition method	Dataset (total)	Species	Classifier	Result
(Charpe et al., 2015)	Microscope	15 images	*Plasmodium*	SVM	97.7% accuracy, 97.4% sensitivity, and 97.7% specificity
(Abbas et al., 2019)	Microscope	74 images	*Plasmodium*	SVM, KNN and NB	96.75% sensitivity and 94.59% specificity
(Uc-Cetina et al., 2013)	Microscope	120 images	*Trypanosoma*	Bayesian	98.3% sensitivity and 84.37% specificity
(Diaz et al., 2009)	Microscope	450 images	*Plasmodium*	SVM	94% sensitivity and 99.7% specificity
(Uc-Cetina et al., 2015)	Microscope	12,936 images	*Trypanosoma*	AdaBoost and SVM	100% sensitivity and 93.25% specificity
(Park et al., 2016)	Microscope	Quantitative phase images of unstained cells	*Plasmodium*	LDC, k-NNC and LR	The highest accuracy of 99.7%, 99.5% and 99.1% in LDC, NNC, and LR, respectively
(Liang et al., 2016)	Microscope	27,578 images	*Plasmodium*	CNN	97.37% accuracy, 96.99% sensitivity, 97.75% specificity, and 97.36% F1-score
(Rajaraman et al., 2018)	Microscope	27,558 images	*Plasmodium*	CNN	98.6% accuracy, 98.1% sensitivity, 99.2% specificity, 98.7% F1-score, and 97.2% MCC
(Umer et al., 2020)	Microscope	27,558 images	*Plasmodium*	CNN	99.6% accuracy, 100% precision, 99.92% recall, and 99.96% F1-score
(Luo et al., 2021)	Imaging flow cytometry	80,146 images	*Cryptosporidium and Giardia*	CNN	> 99.6% accuracy, 97.37% sensitivity and 99.95% specificity
(Li et al., 2020a)	Microscope	13,135 images (T400 dataset) and 14,992 images (T1000 dataset)	*Toxoplasma*	Transfer learning	T400 –93.1% accuracy, 93.9% F1-score, 96% recall, and 91.9% precision; T1000 –94.0% accuracy, 93.9% F1-score, 92.9% recall, and 94.9% precision
(Li et al., 2020b)	Microscope	24,358 images	*Toxoplasma, Plasmodium and Babesia*	Deep cycle transfer learning	95.7% accuracy, 95.7% F1-score, 95.7% recall, and 95.8% precision
(Li et al., 2021)	Microscope	79,672 images	*Plasmodium*	GCN	98.3% accuracy, 98.5% precision, 98.3% recall, and 98.3% F1-score

**Table 3:** Available tools for microscopic image recognition and detection of protozoal pathogens.

Model	Description	Species	Availability	Refs
CLoDSA	An image augmentation library for object classification, localization, detection, semantic segmentation and instance segmentation.	*Plasmodium*	https://github.com/joheras/CLoDSA	(Casado-Garcia et al., 2019)
R-CNN	Automated cell identification of malaria parasite cells using Region-based convolutional neural network model for both brightfield and fluorescence images.	*Plasmodium*	https://github.com/broadinstitute/keras-rcnn	(Hung et al., 2020)
DTGCN	A tool based on GCN was used for recognizing blood smear images of malaria parasite on multi-stages.	*Plasmodium*	https://github.com/senli2018/DTGCN_2021	(Li et al., 2021)
DCTL	Detection of three apicomplexan parasites by employing deep cycle transfer learning method to conduct microscopic image analysis.	*Toxoplasma*, *Plasmodium* and *Babesia*	https://github.com/senli2018/DCTL	(Li et al., 2020b)
FCGAN	A microscopic image recognition method by employing fuzzy cycle generative adversarial network by the combination of transfer learning.	*Toxoplasma*	https://github.com/senli2018/FCGAN/	(Li et al., 2020a)
MCellNet	A deep neural network processing pipeline by combining the imaging flow cytometry as a detection system realizes rapid, accurate and high-throughput detection and classification with respects to the waterborne parasites.	*Cryptosporidium and Giardia*	https://github.com/upeluo/mcellnet	(Luo et al., 2021)

#### *Plasmodium* spp.

There are five major *Plasmodium* species (*Plasmodium falciparum*, *P. vivax*, *P. ovale*, *P. malaria* and *P. knowlesi*) that have the ability to infect humans (Sherling and Van Ooij, 2016). Of these, *P. falciparum* and *P. vivax* are the two most common *Plasmodium* parasites due to their wide prevalence and infection worldwide. In the cases of human infection, *Plasmodium* parasites exhibit essentially the same but complex lifecycle stages that involve two major hosts, i.e., a vertebrate host (human) and a vector host (mosquito), of which intraerythrocytic stages (trophozoite, schizont, and gametocyte stages) cause malaria. Since the intraerythrocytic stages vary significantly in morphology, the different stages of this parasite can be recognized easily by stained blood smear images, which can serve as the image sets used for ML-based diagnosis analysis. In terms of malaria diagnosis, some critical solutions in ML, including image collection, image preprocessing, parasite and cell segmentation, feature selection, feature extraction, and cell classification, have been reviewed previously by Poostchi et al. (2018).

An SVM method based on the watershed threshold algorithm for the detection of the lifecycle stage in microscopic blood images was proposed in Charpe et al. (2015) and shows 97.7% accuracy, 97.4% sensitivity, and 97.7% specificity. Abbas et al. (2019) implemented the classification of the lifecycle stage of malaria parasites in blood smear images using multiclass SVM, k-NNC and NB algorithms, of which the multiclass SVM can generate the best classification results by incorporating histograms of oriented gradients and local binary pattern features, yielding 96.75% sensitivity and 94.59% specificity based on the proposed framework described by the authors (Abbas et al., 2019). A study in (Diaz et al., 2009) used SVM to classify blood smear images and to detect infected erythrocytes, which also achieved good performance in sensitivity (94%) and specificity (99.7%). In addition to the excellent performance of the SVM method, Park et al. (2016) utilized quantitative phase images of unstained cells and three ML algorithms (including LDC, LR, and k-NNC) to detect *P. falciparum* parasites at the trophozoite and schizont stages, which achieved accuracies of 99.7% in detecting the schizont stage (LDC method) and 98% and 99.5% for discriminating early trophozoites (or ring stage) (LDC method) and late trophozoites (k-NNC method), respectively.

**Figure 2:**
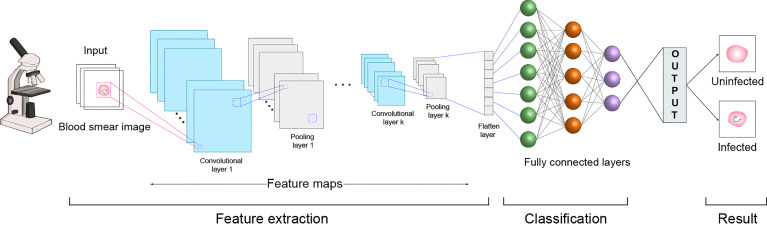
Parasite recognition and detection in blood smear image using CNN approach. A blood smear image for intracellular parasite infection is a typical microscope image, which allows CNN to take in an input microscope image, assign importance (learnable weights and biases) to various aspects and be able to accurately differentiate a parasite-infected red blood cell from a normal red blood cell or discerning different lifecycle stage of a parasite. The feedforward layers of CNN contain the input layer, convolutional layer, pooling layer, flatten layer, and fully connected layer. A three-dimensional matrix presents the image data contained in the input layer, and the image is reshaped into a single column. To conduct the convolution operation, the layer is used to create several smaller picture windows to deliver data information. Pooling is a down-sampling operation capable of reducing the dimensionality of the feature map. The flatten layer is used to “flatten” the input, that is, making the multi-dimensional input into one-dimensional data. The fully connected layer is used to identify and classify the object of an image, thereby obtaining output results for probabilistic detection in red blood cells.

In terms of these conventional ML algorithms, particularly the commonly used SVM, superior performances may be achieved in a classification task for a relatively small number of image sets; however, novel systems are still needed to produce highly scalable and superior results when processing a larger set of images. As an emerging and important form of ML, DL algorithms exhibit exceptional traits for larger digital image recognition and analysis (Wu and Chen, 2015; Ker et al., 2017), although it generally requires high computing power and massive image datasets. In DL architectures, CNN is one of the most successful approaches due to its significant capability in computer vision and image processing (Nebauer, 1998). CNN is based on a series of convolutional and pooling layers to process image that has a grid pattern, and have become the most common existing approaches for image classification between parasite infected and uninfected cells (**Figure 2**).

In previous studies, more than twenty articles have reported CNN applications in the detection of malaria parasites. We herein searched the references of selected articles to present CNN applications. For example, for the first DL application in parasitic diseases, Liang et al. (2016) designed a 16-layer CNN toward recognizing and classifying malaria parasites, achieving 97.37% accuracy, 96.99% sensitivity, 97.75% specificity, and 97.63% F1-score. Rajaraman et al. (2018) used five pretrained CNN (AlexNet, VGG-16, ResNet-50, Xception, and DenseNet-121) as extractors and then evaluated these models through classification based on 27,558 cell images with equal instances of parasitized and uninfected cells (i.e., end-to-end feature extraction and classification); the authors also observed that the ResNet-50 model achieved optimal results for diagnosis at the cell level, resulting in 98.6% accuracy, 98.1% sensitivity, 99.2% specificity, and 98.7% F1-score. Umer et al. (2020) proposed a stacked CNN architecture (the difference from Rajaraman et al. (2018) is the designed number of layers and the size of the kernel) for automatic detection of malaria parasites using 27,558 cell images, which improved the performance after five-fold cross-validation with 99.96% accuracy, 100% precision, 99.92% recall and 99.96% F1-score. In addition to CNN methods, Li et al. (2021) recently developed a novel DL method based on a graph convolutional network called DTGCN model to classify multistage parasitized and uninfected cells (including ring, trophozoite, schizont, and gametocyte stages) with a total of 79,672 single-cell images, which achieved 98.3% accuracy, 98.5% precision, 98.3% recall, and 98.3% F1-score. Collectively, these existing DL approaches have shown promising results for malaria parasite detection, which can be attributed to the plasticity of DL architectures and the availability of mass-produced cell image sets available from public medical libraries.

Mobile device systems such as smartphone applications combining ML models and utilizing microscopic images as an object for analysis are expected to provide applicable value for parasite detection. Fuhad et al. (2020) proposed a model utilizing DL-based methods to detect malarial parasites from microscopic images on smartphones with an accuracy of 99.23%. Yang et al. (2020) developed a customized CNN model and implemented it on smartphones to detect malaria parasites with over 93% accuracy. Yu et al. (2020) designed an Android mobile phone application named Malaria Screener (Available on Google Play), which makes smartphones capable of automated malaria diagnosis under light microscopy, including image acquisition, screening, and management. Davidson et al. (2021) used a pretrained Faster Region-based CNN model to detect malaria infection and stage of malaria parasites from camera phone images with an average precision of 99%, and provided an online web tool (called PlasmoCount available at https://www.baumlab.com/plasmocount) that can be used by the malaria research community. Collectively, these applications can provide great help to reduce the clinician’s labor and, through eliminating the need for highly trained personnel, can also serve as an important adjuvant diagnostic tool to improve point-of-care diagnosis in resource-limited places.

#### Other Protozoan Parasites

*Toxoplasma* is also a parasite that has attracted much attention from researchers because it infects almost all warm-blooded vertebrates and has multiple divergent life cycle stages. Two lifecycle stages – tachyzoites (invading red blood cells) and tissue cysts (invading brain or muscle tissue) – are correlated with the intermediate host, while another stage – the oocyst – is linked to the felid host and is released by feces into the external environment (Tenter et al., 2000). Tachyzoites are generally crescent or banana-shaped in microscope images and are an important stage for acute toxoplasmosis diagnosis, as they allow disease treatment and control. Based on the features of the *Toxoplasma* life cycle, Li et al. (2020a) developed a transfer learning-based microscopic image recognition approach to identify *Toxoplasma* tachyzoites on × 400 (T400 dataset) and ×1,000 (T1000 dataset) images with a total of 28,127 single-cell images by comparing multiple DL models, which achieved the classification of banana-shaped *Toxoplasma* with accuracy of > 93% in both the T400 and T1000 datasets. In addition, Li et al. (2020b) also proposed a transfer learning method to compare the classification of apicomplexan parasites, including *Toxoplasma* and two other protozoan parasites (*Plasmodium* and pear-shaped *Babesia*), obtaining an average accuracy of 95.7% and an average AUC of 99.5% for all parasite types.

*Cryptosporidium* and *Giardia* are two common parasites of infectious enteritis in humans and agricultural animals (e.g., cattle and water buffalo), with widely documented waterborne outbreaks worldwide (Feng and Xiao, 2011; Bouzid et al., 2013; Abeywardena et al., 2015). Compared to other Apicomplexan protozoans, the lifecycle of *Cryptosporidium* and *Giardia* is relatively simple. Among them, *Cryptosporidium* involves infectious oocysts released by the infected host through feces into the public environment and includes several intra-host stages from asexual to sexual reproduction (Bones et al., 2019); *Giardia* has two morphological stages, namely, the intra-host trophozoite and the environmentally resistant cyst (an infectious stage). Infection can be acquired following the ingestion of water and food contaminated with the infectious stage oocysts (*Cryptosporidium*) or cysts (*Giardia*), which results in the host’s gastrointestinal diseases and various inflammations (Abeywardena et al., 2015). From a public health perspective, owing to the great zoonotic impact of *Cryptosporidium* and *Giardia* on human health (Cacciò et al., 2005; Abeywardena et al., 2015), the detection of infectious stages (oocyst and cyst) in the environment bears particularly critical significance for the prevention and control of infection. In an early study, Widmer et al. (2002) used ANN to detect immunofluorescently labeled *Cryptosporidium* oocysts (525 images in total). Similarly, Widmer et al. (2005) utilized ANN methods to identify *Cryptosporidium* oocysts (1,586 images) and *Giardia* cysts (2,431 images) in a relatively large-scale image set, and the correct rates of detected oocysts and cysts were calculated to be 91.8% and 99.6%, respectively. With the development of technology for bioparticle images such as imaging flow cytometry and its advantage in capture speed, Luo et al. (2021) recently reported a DL-enabled high-throughput system called MCellNet, which is used for *Cryptosporidium* and *Giardia* detection in drinking water. This system was tested using 80,146 single-cell images that were rapidly acquired by imaging flow cytometry, showing > 99.6% accuracy, 97.37% sensitivity, and 99.95% specificity.

In terms of other protozoan parasites, however, currently, there are no available applications utilized on smartphone or web-based systems. Given that some pathogenic parasites belonging to the class of ubiquitous microorganisms exist in our living environment, particularly water-borne and food-borne parasites (e.g., *Toxoplasma*, *Cryptosporidium*, and *Giardia*) that, through oral infection, potentially threaten human health, it is worth looking forward in the future to develop intuitive, convenient and easy-to-use applications for parasite detection in the environment.

### Public Health Surveillance

Public health surveillance is the systematic collection, analysis, and dissemination of data on diseases of public health importance in order to take proper actions to prevent or stop the further spread of diseases (Nsubuga et al., 2006). Traditional approaches for public health surveillance mainly depend on the usage of mathematical statistics (Sonesson and Bock, 2003; Höhle et al., 2009). However, with the tremendous growth of AI-derived techniques in recent years, those based on ML methods can directly derive models to perform regression, classification, and time-series analyses and to implement public health surveillance, instead of relying only on stringent statistical techniques for the data-generating system. This makes this approach more effective to solve uncertain and nonlinear problems in some complex applications.

**Figure 3:**
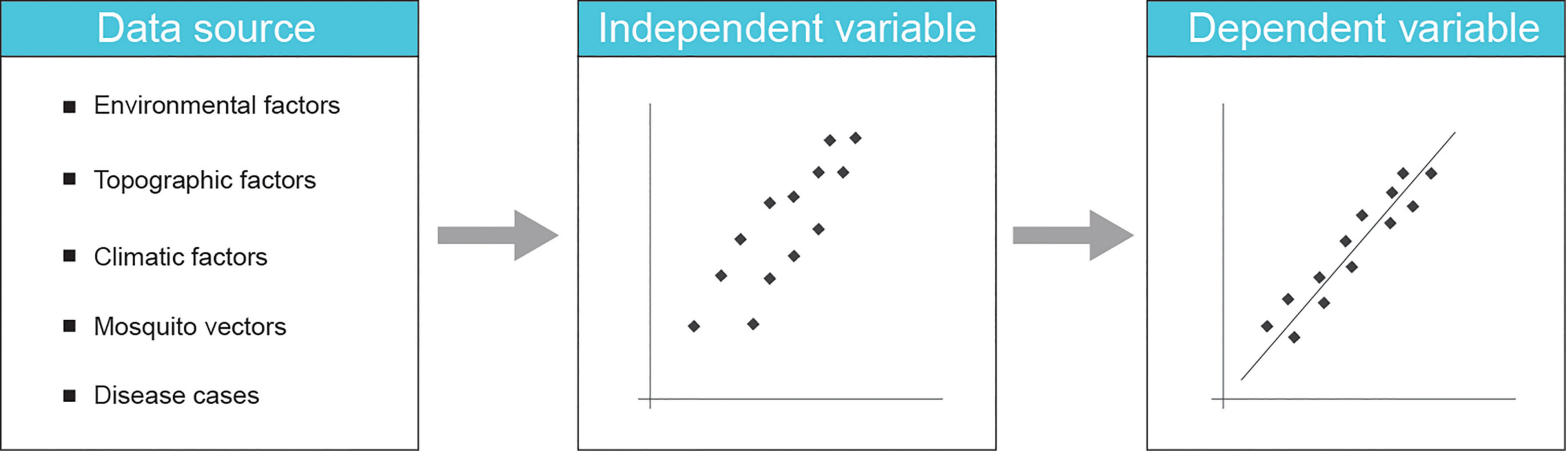
In terms of public health surveillance in protozoal diseases, a variety of data types can be used to construct machine learning models, capable of deriving data from independent variables to dependent variables to obtain the prediction result of disease.

ML algorithms have enabled the utilization of AI to detect epidemiological changes, trace disease outbreaks, and analyze disease trends and risks, from public health surveillance data sources to provide early warning, targeted interventions, and control measures (Zeng et al., 2021). Disparate types of data sources for public health surveillance often involve complex and heterogeneous factors, which are essential for identifying early, accurate, and reliable signals of disease outbreaks. With these existing applications, a variety of data sources for implementation of ML-enhanced public health surveillance of protozoal diseases, particularly malaria, can be derived from the following five major aspects (**Figure 3**): environmental factors (e.g., temperature, rainfall, and relative humidity) (Kiang et al., 2006; Ye et al., 2007; Castro, 2017), topographic factors (e.g., elevation, slope, aspect, and ruggedness) (Cohen et al., 2010; Atieli et al., 2011), geographical factors (e.g., nationality and region) (Zhou et al., 2010; Ayele et al., 2012), vector transmissions [e.g., the population of mosquito vectors for malaria parasites (Athrey et al., 2012) and the population of insect vectors for Chagas disease (Justi and Galvao, 2017)], and disease case reports [e.g., patient clinical information, signs and symptoms (Lee et al., 2021; Yadav et al., 2021)]. One or several operationalizable sources of data that contain valuable signals can be chosen as features, thereby testing signals on the ML model to predict the disease transmission dynamics and to evaluate the public health potential.

Malaria is still the main infectious disease of concern to scientists, as it has considerable significance for public health in many tropical and subtropical areas of the world. By taking advantage of various data resources, different malaria models have been developed to predict malaria transmission. According to the reports in recent years, for instance, Haddawy et al. (2018) developed a predictive model using Bayesian networks to predict malaria outbreaks on the basis of weekly infection cases and environmental covariates, such as rainfall, temperature and vegetation, offering good predictive capability during numeric case and outbreak predictions. Thakur and Dharavath (2019) proposed a predictive model for local malaria prevalence on the basis of the ANN method by collecting clinical and environmental variables with big data in local areas, as well as various satellite data that include rainfall, relative humidity, temperature and vegetation from the time period of 1995-2014. Based on parasite infection reports, Lee et al. (2021) extracted patient information obtained from PubMed abstracts and utilized six ML models (SVM, RF, multilayered perceptron, AdaBoost, gradient boosting, and CatBoost) to predict malaria: all models exceeded 90% accuracy, indicating that nationality and region of travel are important factors to diagnose malaria.

Transmission vectors also play a crucial role in the prediction of protozoal diseases. For instance, correctly identifying insect vectors of Chagas disease in digital images employing DL algorithm has shown benefits for people who are without entomological expertise (Khalighifar et al., 2019); in a malaria example, because this disease is transmitted *via* the bite of infected female *Anopheles* mosquitoes that contain *Plasmodium* parasites, it has demonstrated potential for estimating the parity status of wild mosquitoes using an autoencoder and ANN-based method (Milali et al., 2020). These surveys concerning transmission vectors combined with ML methods collectively contribute to the identification of transmission vectors and monitoring of disease prevalence to indirectly support public health surveillance.

Different protozoan parasites bear divergence in genetics, geographical distribution, lifecycle, host, pathogenicity, and so on. Thus, the implementation of public health surveillance using ML methods and relative prediction strategies is not completely consistent among different protozoal diseases, and the parasite’s life cycle and parasitic manner must be considered when choosing appropriate data types.

### Host-Parasite Interaction

To establish successful pathogenicity, protozoan parasites can infect different host tissues and cell types, and have the ability to evolve their strategies to escape the immune response of the host (Cox, 2002). One effective strategy for a parasite is to secrete virulence effectors into host cells to subvert various host pathways (Hunter and Sibley, 2012; Draper et al., 2018). In general, a successful method of infection is mainly through PPI where the parasite proteins target the host proteins, which is capable of forming a biological network (Dallas et al., 2017). During the processes of infection and invasion, PPI are crucial for a parasite to initiate infection and establish a host immune escape mechanism and are substantially implicated in the identification of potential targets for new and effective therapeutics. In most molecular experiments, identifying host-parasite PPI is time-consuming, expensive, and generally dependent on the experimental experience of researchers.

**Figure 4:**
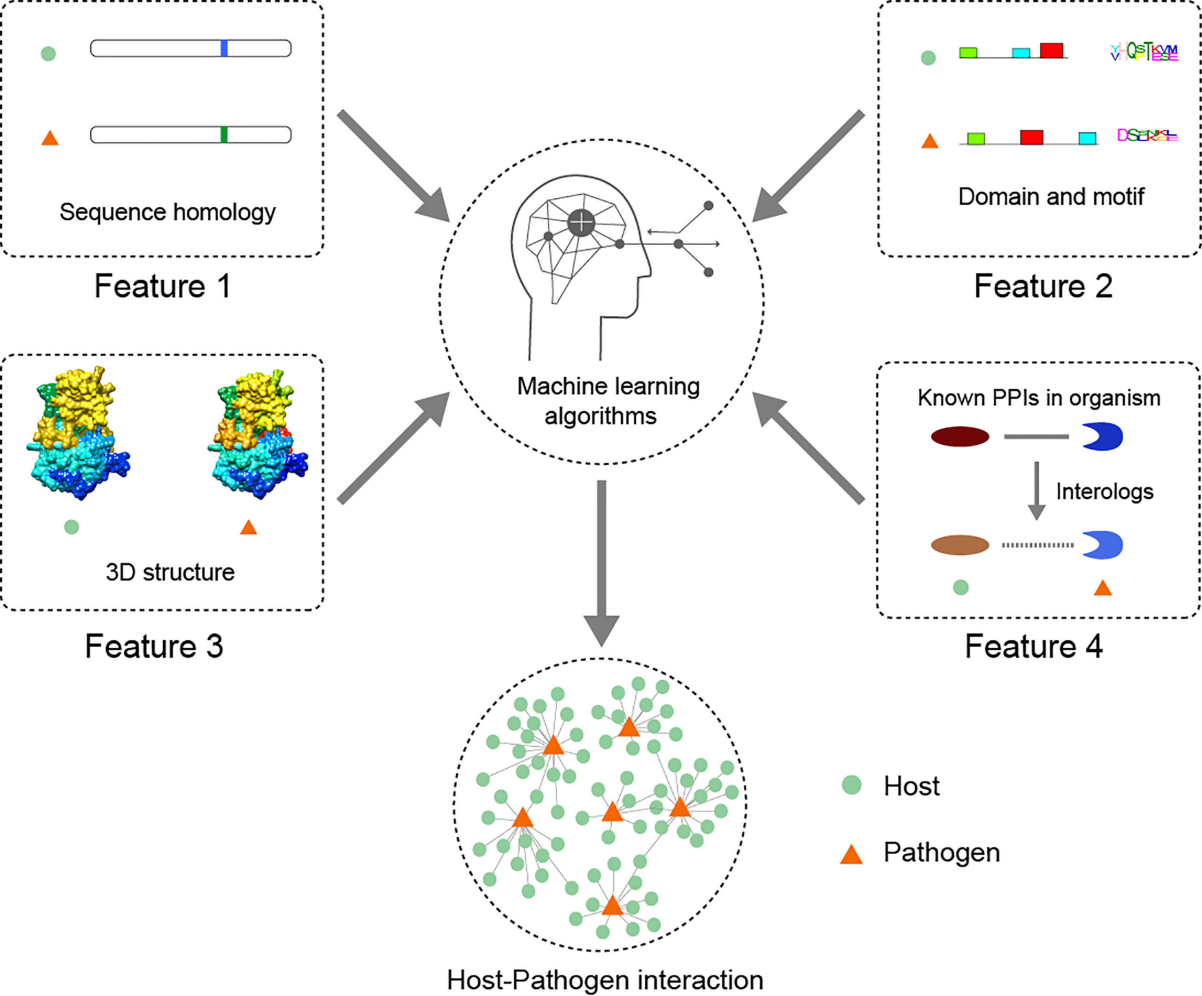
In host-parasite interaction, four common features in a computational workflow are worth considering for which they can further improve the accuracy of prediction, including protein 3D structural information, domain-domain interaction and/or domain-motif interaction, and interologs based on the known PPI in an organism.

Interspecies interactions between hosts and pathogens, including host-protozoan PPI, have long been explored by employing various computational methods (Mariano and Wuchty, 2017; Soyemi et al., 2018). Prediction methods require features extracted from PPI to learn. Four main features are the crucial components of bioinformatics analysis in facilitating the construction and prediction of host-pathogen PPI (**Figure 4**): (i) sequence homology-based methods (Murakami and Mizuguchi, 2014; Zhou et al., 2014), (ii) domain and motif-based methods (including domain-domain interaction and domain-motif interaction) (Wojcik and Schachter, 2001; Kshirsagar et al., 2013; Segura-Cabrera et al., 2013; Zhou et al., 2013; Becerra et al., 2017), (iii) 3D structure-based methods (Davis et al., 2007; Doolittle and Gomez, 2010; De Chassey et al., 2013), and (iv) transferring known PPI from the same organism based on the similarity of sequence homology, domain and motif into the predictive host-pathogen PPI (i.e., interologs) (Wuchty, 2011; Nourani et al., 2015). Additionally, further computational investigation for potential interactions using other relative features, such as biological function, evolutionary information, cellular localization, and expression profile data, can also discard some false-positive interactions and immensely improve the quality of interaction candidates (Mariano and Wuchty, 2017).

ML methods have been adapted to predict possible PPI between hosts and parasites. Previously, Dyer et al. (2007) proposed a Bayesian approach for integrating known intraspecies PPI with protein domain profiles to predict the interactions between *P. falciparum* and the human host, producing 516 PPI between proteins from these two organisms. Wuchty (2011) utilized a computational method to infer homologous and conserved protein interactions between *P. falciparum* and the human host and evaluated them by employing the RF algorithm. Additionally, the author further filtered the false-positive based on expression profiles and molecular traits, pooling a total of 2,244 host-parasite PPI. Ghedira et al. (2020) developed an integrative computational approach through a combination of omics expression data (composed of infected human red blood cells and *P. falciparum* protein expression profiles), domain- and structure-based PPI, similarity of gene ontology, and eight ML classifiers (i.e., k-NNC, logistic regression, decision tree, RF, Adaboost, voting classifier, NB and SVM) to predict PPI between *P. falciparum* and the human host, reporting 716 protein interactions. In a recent study, Suratanee et al. (2021) predicted human-*P. vivax* protein associations based on multiple features, including known protein-protein networks in a single organism in humans or *P. vivax* and protein sequence similarity, and employed four ML algorithms (NB, neural network, RF, and SVM) to classify defined and undefined associations. All these methods that predict human-*Plasmodium* PPI through supervised learning require the combination of protein sequences and other relative protein sequence information to sever as appropriate positive and negative training sets to robustly classify the interacting proteins. These predicted candidates from a list of host-parasite PPI could provide novel promising targets for wet-experimental validation, thereby reducing time and development costs.

Apart from the prediction of host-parasite PPI at the protein level, phenotypic image analysis based on machine intelligence algorithms can also be employed with respect to cell image sets to recognize variations in cell properties and to analyze interactions between biological systems (Smith et al., 2018). For example, Fisch et al. (2019) developed an image-based analysis platform called HRMAn that incorporates decision tree classification and deep CNN to analyze the infection of cells with intracellular *Toxoplasma*. Additionally, the authors elaborated the capability of HRMAn to learn phenotypes from image sets thereby analyzing the host response and parasite fate at the single-cell level (available at https://hrman.org/).

### Drug Discovery

The rapid emergence of drug resistance and genetic variation of parasite strains that make antibiotic drugs less effective for treatment emphasizes the urgent need to develop novel drugs (Borst and Ouellette, 1995; Su et al., 2019). Drug discovery starts with the identification of an effective compound and its binding affinity with a target protein. Additionally, the identified compounds should have bioactive ability to limit or block parasite growth and reproduction within the infecting host; at the same time, one of the key issues addressed is to reduce toxic and off-target effects. Nevertheless, in most cases, drug discovery is a considerably complex and long process, ranging from target selection to drug approval, which generally requires more than ten years (Rifaioglu et al., 2019). With the availability of various high-throughput sequencing data and small-molecule chemical libraries, the following question is posed: how can we utilize computational approaches to find effective compounds and to predict the probability of effective compound-target pairs through various large-scale datasets, while reducing the cost and time period of the early development of innovative drugs?

**Figure 5:**
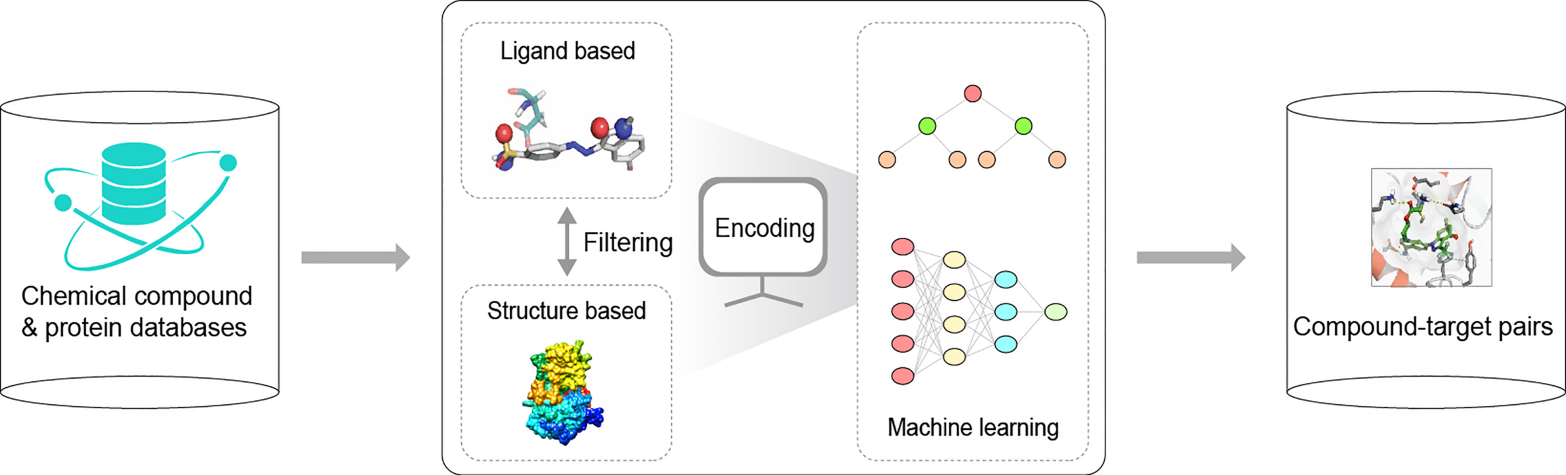
A basic workflow for virtual screening platform in the process of drug discovery. In this process, machine learning represents a powerful tool to predict compound-target pairs using compound datasets and functional known proteins.

Until now, the main method in the drug discovery process has relied on VS (Walters et al., 1998; Kitchen et al., 2004), also known as *in silico* screening, which is often assisted by computational methods, including ML methods. With respect to a disease, the goal of VS is to find the most promising compound assays from large chemical databases [e.g., PubChem (Kim et al., 2016), ChEMBL (Bento et al., 2014), DrugBank (Law et al., 2014), and ZINC (Sterling and Irwin, 2015)], and to identify optimal compound-target pairs based on known annotations in protein databases [e.g., UniProtKB (UniProt Consortium, 2015), InterPro (Finn et al., 2017), and Pfam (Finn et al., 2016)]. Previous reviews (Lo et al., 2018; Rifaioglu et al., 2019; Kimber et al., 2021) have detailed the application and development of ML in VS for drug discovery. Moreover, by leveraging computer-aided encoding in VS pipelines, ML techniques can be employed in structure-based VS and ligand-based VS (Wilson and Lill, 2011; Vázquez et al., 2020). Structure-based VS uses the 3D structure of both ligand and target to predict their binding affinity, while ligand-based VS only requires ligand properties, such as molecular fingerprints or descriptors (Cereto-Massague et al., 2015) that encode their structural characteristics as vectors, to identify the similarity between a test compound and a known active compound of a target. More information on cheminformatics studies related to structure-based and ligand-based VS can be found in previous reviews, such as in (Wilson and Lill, 2011; Vázquez et al., 2020). Here, a brief workflow for the ML-based VS platform in bioinformatics is shown in **Figure 5**.

Likewise, the so-called VS-based methods including a combination of ML models are also widely applied to the research of drug discovery against protozoal diseases, such as malaria (Kumari and Chandra, 2015; Urista et al., 2020) and Chagas disease (Ekins et al., 2015; De Souza et al., 2019; Yasuo et al., 2021). In the ligand-based VS method, ML is usually used for the classification of active and inactive compounds by using appropriate ML algorithms. In terms of anti-malarial compound prediction, for example, an early study by Kumari and Chandra (2015) classified active and inactive compounds based on the known activity score obtained from the PubChem database and trained three classifiers (i.e., RF, NB, and J48) to predict bioactive anti-malarial molecules by inhibiting a target protein of *P. falciparum*, M18AAP – a critical enzyme for the survival of malaria parasites, finding that the RF classifier could perform better classification of compounds (97.94% specificity) than other classifiers. Urista et al. (2020) compared multiple ML algorithms for the prediction of nanoparticle-compound complexes against malaria parasites utilizing chemical compounds from the ChEMBL database and experiment-based nanoparticle data. The authors found that the best-performing algorithm, an RF classifier trained using 27 selected features of drugs and nanoparticles, yielded an AUC of 99.21% in ten-fold cross-validation. In terms of compound prediction in Chagas disease, Ekins et al. (2015) developed Bayesian classifier models that were used to virtually screen compounds from the CDD database (https://www.collaborativedrug.com/), and the top-scoring compounds were tested on an acute Chagas mouse model, with an identified antiparasitic efficacy of 85.2%. In another study of compound prediction for Chagas disease, De Souza et al. (2019) notably investigated 363 structurally diverse compounds using ANN and KPLS algorithms to predict the anti-parasite activity, yielding q^2^ values (correlation coefficient for the test compound set) of 0.81 and 0.84 on the ANN and KPLS models, respectively.

In addition, some studies have demonstrated the abilities of DL models that were used to implement a VS pipeline and to predict compounds against a large number of datasets. Keshavarzi Arshadi et al. (2019) developed a graph convolutional neural network DL model named DeepMalaria to predict the anti-*P. falciparum* inhibitory properties of compounds through a ligand-based VS method, followed by validation of this model by predicting hit compounds from a known compound library and already approved drugs. The authors also further improved this model by using transfer learning and external validation on an independent and imbalanced dataset, showing that most of the predicted active compounds have greater than 50% inhibition of the *P. falciparum* parasite. Similarly, in a recent study, a DL-based VS model proposed by Neves et al. (2020) was used to predict the antiplasmodial activity and cytotoxicity of untested compounds to screen the prioritized compounds through experimental evaluation. These applications highlight the capability of the DL framework in leveraging various large compound datasets to identify antiplasmodial activity, which may be equally applicable to other protozoan parasitic diseases, despite a lack of relevant research reports.

### Omics and Vaccine Discovery

With the advancement of omics-based technologies such as transcriptomics and proteomics studies, it has become increasingly feasible to acquire personalized data about protozoan parasites (Cowell and Winzeler, 2019). ML algorithms promise the ability to excavate these data and incorporate other bioinformatics tools and methods to perform deeper analyses in several settings. In transcriptomics, RNA-seq techniques are widely used to accurately detect gene expression profiles, and the ML method can be used to conduct protein classification based on transcriptomic profiles, such as in malaria parasites (Mitrofanova et al., 2008), or to explore transcriptomic signatures through differential gene expression analysis in parasite-infected hosts such as leishmaniasis patients (Adriaensen et al., 2020). In the context of proteomics, ML has also been used to predict protein complexes with unknown function based on protein correlation profiling mass spectrometry, such as in *T. brucei* (Crozier et al., 2017), or to identify protein and/or peptide vaccine candidates for target parasites, such as *Toxoplasma* and *Plasmodium* (Goodswen et al., 2013b).

As a radical endeavor to prevent infectious disease, vaccine development is a necessary process, which begins with the identification of candidate antigens using computational approaches and follows with the prediction of whether the host inoculated with the vaccine can produce a protective immune response against a given parasite. Typically, parasite-derived effectors, such as virulence factors and outer membrane, invasion, and virulence-related proteins, are potential antigens used for vaccine development: in the vaccine discovery pipeline, they can be effectively predicted by *in silico* approaches (Goodswen et al., 2013a; Goodswen et al., 2014). Although the immunogenicity regarding a set of candidate vaccines cannot be confirmed immediately without experimental validations, many efforts in elucidating a worthy list of vaccine candidates have been made, such as predicting the most promising vaccines against *Toxoplasma* and *Plasmodium* (Goodswen et al., 2013b) and *Babesia* (Goodswen et al., 2021b; Goodswen et al., 2021c) using various ML algorithms, e.g., adaptive boosting, k-NNC, NB, ANN, RF and SVM. These works provide a reference for researchers engaged in vaccine development to conduct further laboratory validation, which will save substantial time and money.

## Conclusion

With the constant development and improvement of ML, it has established its position across the field of infectious diseases, including parasitic protozoans and protozoal diseases. We herein provided a comprehensive review of ML applications in terms of pathogen detection, public health surveillance, host-parasite interaction, drug discovery, omics, and vaccine discovery. Of these, image-based parasite detection has achieved the most significant results in practical applications, particularly the use of DL algorithms. Given these successful cases that detect protozoal pathogens by image recognition and classification, it is feasible that more studies in the future should apply ML techniques to carry out the detection of water- and food-borne protozoans causing environmental pollution and should develop convenient detection tools for public health researchers. In many other applications, although ML methods hold substantial promises, they are still in the exploratory stage and require further development and perspective validation. Some key challenges also exist: for example, the goal of public health surveillance in infectious disease is to predict disease burden and identify disease outbreaks, but ML methods partly depend on data sources of collection, underscoring the requirement for larger and more diverse datasets. Furthermore, ML is capable of performing effective data mining and identifying valuable molecular targets regarding host-parasite interaction, drug and vaccine discovery, but there are some inherent limitations: (i) the functions of the majority of proteins are unknown [e.g., in the annotated genome information, 51.5% of *T. gondii* proteins (ME49 strain, version release 54) and 71.8% of *P. falciparum* proteins (3D7 strain, version release 54) are hypothetical proteins or have unknown functions]; (ii) how to effectively utilize omics data and integrate them into ML prediction models; (iii) lacking adequate experimental validation data as training datasets. Because datasets play critical roles in the process of ML, it is warranted for future studies to combine data from various sources, embrace data sharing, and establish public databases for ML. Particularly, regarding the development of drugs and vaccines, researchers should screen experimentally validated molecule targets (e.g., pharmaceutical compounds, biomacromolecules, and antigenic epitopes), use these data to train ML models with high robustness and accuracy, and develop practical bioinformatics tools for use by microbiologists.

## Author Contributions

Conceptualization: QZ. Writing—Original Draft Preparation: R-SH. Writing—Review and Editing: R-SH, AE-LH, and QZ. Project Administration: QZ. All authors have read and agreed to the published version of the manuscript.

## Funding

The work was supported by the National Natural Science Foundation of China (No. 62131004, No.61922020), the Sichuan Provincial Science Fund for Distinguished Young Scholars (2021JDJQ0025), and the Special Science Foundation of Quzhou (2021D004).

## Conflict of Interest

The authors declare that the research was conducted in the absence of any commercial or financial relationships that could be construed as a potential conflict of interest.

## Publisher’s Note

All claims expressed in this article are solely those of the authors and do not necessarily represent those of their affiliated organizations, or those of the publisher, the editors and the reviewers. Any product that may be evaluated in this article, or claim that may be made by its manufacturer, is not guaranteed or endorsed by the publisher.

## Abbreviations

ML, machine learning; AI, artificial intelligence; DL, deep learning; WHO, world health organization; DNA, deoxyribonucleic acid; RNA, ribonucleic acid; FE, feature extraction; FS, feature selection; MRMD, max-relevance-max-distance; SVM, support vector machine; NB, Naïve Bayes; RF, random forest; k-NNC, k-nearest neighbor classification; LDC, linear discriminant classification; LR, logistic regression; TP, true positive; TN, true negative; FP, false positive; FN, false negative; MCC, Matthews correlation coefficient; ROC, receiver operating characteristic; TPR, true positive rate; FPR, false positive rate; AUC, area under the curve; PCR, polymerase chain reaction; CNN, convolutional neural networks; GCN, graph convolutional network; ANN, artificial neural networks; PPI, protein-protein interactions; VS, virtual screening; KPLS, kernel-based partial least squares regression.
